# Long-term outcomes of nucleus accumbens/anterior limb of the internal capsule deep brain stimulation in treatment-resistant obsessive-compulsive disorder

**DOI:** 10.3389/fpsyt.2026.1905823

**Published:** 2026-10-19

**Authors:** Fátima Ximena Cid-Rodríguez, Rabea Schmahl, René Márquez-Franco, Petra Heiden, Marvin Antonio Soriano-Ursúa, Wencke Wagemann, Daniel Huys, Juan C. Baldermann, Jens Kuhn, Veerle Visser-Vandewalle, Pablo Andrade

**Affiliations:** 1 Department of Stereotactic and Functional Neurosurgery, University of Cologne, Faculty of Medicine and University Hospital Cologne, Cologne, Germany; 2 Graduate Studies and Research Section, Escuela Superior de Medicina, Instituto Politécnico Nacional, Mexico City, Mexico; 3 Department of Psychiatry and Psychotherapy, University of Cologne, Faculty of Medicine and University Hospital Cologne, Cologne, Germany; 4 Department of Psychiatry and Psychotherapy III, LVR Hospital Bonn, Bonn, Germany; 5 Department of Psychiatry and Psychotherapy, Medical Center - University of Freiburg, Faculty of Medicine, University of Freiburg, Freiburg, Germany; 6 Alexianer Hospital Cologne, Alexianer Köln GmbH, Cologne, Germany

**Keywords:** anterior limb of the internal capsule, deep brain stimulation, long-term follow-up, neurosurgery, nucleus accumbens, obsessive-compulsive disorder, Y-BOCS

## Abstract

**Background:**

Deep brain stimulation (DBS) is a therapeutic option for carefully selected patients with severe, treatment-resistant obsessive-compulsive disorder (OCD), but very long-term outcomes remain insufficiently characterized. We evaluated clinical outcomes after DBS targeting the nucleus accumbens/anterior limb of the internal capsule (NAcc/ALIC) over 3–21 years and described the trajectories of early responders, delayed responders, and non-responders.

**Methods:**

This single-center retrospective cohort included 30 consecutive patients (17 women) who underwent NAcc/ALIC DBS between 2004 and 2022. Outcomes were assessed with the Yale–Brown Obsessive Compulsive Scale (Y-BOCS) at baseline (T0), 1 year after surgery (T1), and the latest follow-up at ≥3 years (T2). Response was defined as a ≥35% reduction from baseline. Repeated-measures ANOVA with Bonferroni-adjusted pairwise comparisons was used to assess changes over time.

**Results:**

Y-BOCS scores decreased significantly across T0, T1, and T2. At T2, 22 of 30 patients (73.3%) met the response criterion; eight were delayed responders. Of the 15 patients who met the response criterion at T1, 14 retained it at T2 and 1 did not.

**Conclusion:**

Clinically meaningful improvement was sustained at long-term follow-up, and some patients who had not responded after 1 year met the response criterion later. Once response was achieved, subsequent loss of response appeared uncommon in this cohort. The retrospective design and heterogeneous follow-up duration preclude causal conclusions regarding DBS or the temporal course of improvement.

## Introduction

Deep brain stimulation (DBS) has emerged as a therapeutic option for carefully selected patients with severe, treatment-resistant obsessive-compulsive disorder (OCD). Multiple studies have reported clinically meaningful symptom reductions after stimulation of targets within cortico-striato-thalamo-cortical circuits (1–4). Although long-term cohorts have been published in Amsterdam and in other single-center and multicenter series, the extension over two decades remains uncommon, and timing of response remains incompletely characterized (4–10).

This study evaluated long-term clinical outcomes after DBS targeting the nucleus accumbens/anterior limb of the internal capsule (NAcc/ALIC) in a single-center cohort of patients with treatment-resistant OCD. The primary objective was to characterize change in absolute Y-BOCS scores from baseline to the latest assessment after at least 3 years. Secondary objectives were to describe outcomes at 1 year, interval-specific percentage reductions, baseline-severity subgroups, and the trajectories of early responders, delayed responders, and patients without long-term response. The maximum available follow-up was 21 years.

## Methods

We conducted a single-center retrospective cohort study of consecutive patients with treatment-resistant OCD who underwent DBS at the Department of Stereotactic and Functional Neurosurgery, University Hospital Cologne, between 2004 and 2022. Clinical data were extracted in December 2025. The manuscript was prepared in accordance with the STROBE reporting guidelines for cohort studies. The present study comprises the complete cohort of patients treated during the study period. While some patients participated in previously published clinical trials, their outcomes have not previously been reported with the extended follow-up available in the present study. In addition, the cohort includes patients treated outside formal clinical trials whose clinical outcomes have not been previously reported.

Eligible patients had received continued treatment and follow-up in our department for at least 3 years and had available Y-BOCS scores at baseline, 1 year, and the latest follow-up (from 3 to 21 years). Clinical eligibility for DBS had been determined by a multidisciplinary team and required chronic, functionally disabling OCD with insufficient response to established pharmacological and psychotherapeutic treatments. Detailed historical information on the dose and duration of previous treatment trials was not uniformly available for this retrospective cohort. A total of 34 patients underwent surgery during the study period. Four patients (three women and one man) were excluded because 1-year Y-BOCS data were unavailable after they continued treatment elsewhere. The remaining 30 patients had Y-BOCS data at T0, T1, and T2 and were included in the complete-case analysis.

The combined NAcc/ALIC region was targeted using standard stereotactic coordinates (X = 7–8 mm, Y = 5 mm anterior to the anterior commissure, and Z=−4 mm). Coordinates were individualized to each patient’s anatomy and adjusted to provide a safe trajectory that avoided vascular structures, cortical sulci, and the ventricular system. Lead placement aimed to position one contact within the NAcc, one at the NAcc/ALIC transition, and the two most dorsal contacts within the ALIC. Leads were implanted bilaterally in 25 patients (83.3%) and unilaterally on the right side in five patients (16.7%). Electrode location was verified by postoperative computed tomography. The earliest documented postoperative stimulation settings and the settings recorded at the latest clinical assessment are presented in **Table 1**.

**Table 1:** Demographic, clinical, electrode, and stimulation characteristics of the study cohort.

Patient and response category		Sex	Age at surgery	Follow-up (years)	Electrode	Oldest stimulation parameter	Latest stimulation parameter	Y-BOCS baseline (T0)	Y-BOCS (T1)	Y-BOCS reduction (%) (T0-T1)	Y-BOCS (T2)	Y-BOCS reduction (%) (T0-T2)
Delayed responder	1	F	30	21	Unilateral (R)	R: 0-, 1-, 2-, C+, 90 µs 130 Hz, 6.5 V	R: 1-, 2-, C + 120 µs, 140 Hz, 3.2 V	Extreme (38)	26	31.6	24	36.8
	2	M	44	9	Bilateral	L: 1-, 2-, C+, 120 µs, 130 Hz, 5.0V R: 10-, 11-, G+, 120 µs, 130 Hz, 5.0 V	L: 2-, 3-, G+, 150 µs, 150 Hz, 5.5V R: 10-, 11-, G+, 150 µs, 150 Hz, 5.5 V	Extreme (37)	27	27	20	45.9
	3	F	54	9	Bilateral	L: 2-, 3-, G+, 90 µs, 130 Hz, 4.2V R: 10-, 11-, G+, 90 µs, 130 Hz, 4.2 V	L: 2-, 3-, G+, 110 µs, 130 Hz, 4.2V R: 10-, 11-, G+, 110 µs, 130 Hz, 4.2 V	Extreme (34)	29	14.7	19	44.1
	4	M	43	21	Unilateral (R)	R: 0-, 1-, 2-, 3-, G+, 90 µs, 130 Hz, 4.5 V	R: 0-, 1-, 2-, 3-, G+, 90 µs, 130 Hz, 10.0 V	Extreme (40)	35	12.5	2	95
	5	F	39	18	Bilateral	L: 2-, 3-, G+, 150 µs, 120 Hz 5.0 V R: 6-, 7-, G+, 150 µs, 120 Hz, 5.0 V	L: 2-, 3-, G+, 210 µs, 80 Hz 3.1 mA R: 6-, G+, 210 µs, 80 Hz, 3.6 mA	Extreme (34)	37	-8.8 α	22	35.3
	6	F	35	20	Unilateral (R)	R: 1-, 2-, G+, 120 µs, 140Hz, 6.6 V	R: 2-, (1.6 mA), 3- (1.8 mA), G+; 120 µs, 140 Hz	Severe (29)	20	31	13	55.2
	7	F	50	4	Bilateral	L: 1-, 2-, G+, 90 µs, 130 Hz, 3.5 mA R: 9-, 10-, G+, 90 µs, 130 Hz, 3.5 mA	L: 1-, 2-, G+, 100 µs, 130 Hz, 6.0 V R: 9-, 10-, G+, 100 µs, 130 Hz, 6.0 V	Severe (27)	26	3.7	16	40.7
	8	M	41	3	Bilateral	L: 1-, 2-, G+, 110 µs, 125 Hz, 8.0 mA R: 9-, 10-, G+, 110 µs, 125 Hz 8.0 mA	L: 1-, 2-, G+, 90 µs, 130 Hz, 5.0 mA R: 9-, 10-, G+, 90 µs, 130 Hz 5.0 mA	Moderate (18)	12	33.3	10	44.4
Early and maintained response	9	M	56	8	Bilateral	L: 2-, 3-, G+, 60 µs, 130 Hz, 3.0 V R: 10-, 11-, G+, 60 µs, 130 Hz, 3.0 V	L: 2-, 3-, G+, 60 µs, 130 Hz, 3.5 V R: 10-, 11-, G+, 60 µs, 130 Hz, 3.5 V	Extreme (32)	11	65.6	11	65.6
	10	F	36	3	Bilateral	L: 1-, 2-, G+, 60 µs, 130 Hz, 3.6 mA. R: 9-, 10-, G+, 60 µs, 130 Hz, 3.6 mA	L: 1-, 2-, G+, 60 µs, 130 Hz, 3.6 mA R: 9-, 10-, G+, 60 µs, 130 Hz, 3.6 mA	Extreme (40)	18	55	18	55
	11	F	47	6	Bilateral	L: 1-, 2, 7-, 8-, G+, 90 µs, 130 Hz, 8.0 mA R: 9-, 10-, 15-, 16-, G+, 90 µs, 130 Hz, 8.0 mA	L: 1-, 2-, G+, 100 µs, 130 Hz, 12.0 mA R: 1-, 2-, G+, 100 µs, 130 Hz, 12.0 mA	Extreme (33)	16	51.5	2	93.9
	12	M	22	14	Bilateral	L: 2-, 3-, G+, 130 Hz, 90 µs, 2.5 V. R: 10-, 11-, G+, 90 µs, 130 Hz, 2.5 V	L: 2-, 3-, G+, 130 Hz, 120 µs, 3.5 V. R: 10-, 11-, G+, 120 µs, 130 Hz, 3.5 V	Extreme (33)	17	48.5	5	84.8
	13	M	47	4	Bilateral	L: 1-, 2-, G+, 60 µs, 130 Hz, 4.0mA R: 9-, 10-, G+, 60 µs, 130 Hz, 4.0 mA	L: 0-, 1-, G+, 90 µs, 130 Hz, 6.3 mA R: 8-, 9-, G+, 90 µs, 130 Hz, 6.3 mA	Extreme (37)	21	43.2	11	70.3
	14	M	36	4	Bilateral	L: 1-, G+, 60 µs, 130 Hz, 3,5 – 0.5 mA R: 9-, G+, 60 µs, 130 Hz, 0.5mA	L: 1-, 2-, G+, 250 µs, 130 Hz, 3,5 – 4.0 V R: 9-, 10-, G+, 250 µs, 130 Hz, 5.0mA	Extreme (33)	19	42.4	11	66.7
	15	F	32	15	Bilateral	L: 1-, 2-, G+, 130 Hz, 150 µs, 5.5 V. R: 9-, 10-, G+, 130 Hz, 150 µs, 5.5 V	L: 1-, 2-, G+, 150 µs, 130 Hz 4.0 mA R: 9-, 10-, G+, 150 µs, 130 Hz 4.5 mA	Extreme (35)	22	37.1	19	45.7
	16	F	35	11	Bilateral	L: 1-, 2-, G+, 125 µs, 130 Hz, 3.0 mA R: 8-, 9-, G+, 125 µs, 130 Hz, 3.0 mA	L: 1-, 2-, G+, 125 µs, 130 Hz, 3.0 mA R: 8-, 9-, G+, 125 µs, 130 Hz, 3.0 mA	Severe (30)	7	76.7	3	90
	17	M	44	21	Unilateral (R)	R: 0-, 1-, 2-, 3-, G+, 90 µs 140 Hz, 8.0 V	R: 0-, 1-, 2-, G+, 90 µs, 140 Hz, 7.0 mA	Severe (27)	12	55.6	1	96.3
	18	M	47	9	Bilateral	L: 2-, 3-, G+, 120 µs, 130 Hz, 4.1V R: 10-, 11-, G+, 120 µs, 130 Hz, 4.1 V	L: 2-, 3-, G+, 120 µs, 130 Hz, 4.8V R: 10-, 11-, G+, 120 µs, 130 Hz, 4.8 V	Severe (28)	13	53.6	0	100
	19	F	28	10	Bilateral	L: 1-, 2-, 3-, G+, 80 µs, 150 Hz, 4.8 V R: 10-, 11-, 12-, G+, 90 µs, 150 Hz, 6.0 V	L: 1-, 2-, 3-, G+, 80 µs, 150 Hz, 4.8 V R: 10-, 11-, 12-, G+, 90 µs, 150 Hz, 6.0 V	Severe (27)	14	48.1	1	96.3
	20	F	64	5	Bilateral	L: 0-, 1-, G+, 60 µs, 130 Hz, 3.7 mA R: 9-, 10-, G+, 60 µs, 130 Hz, 3.7 mA	L: 0-, 1-, G+, 90 µs, 130 Hz, 5.0V R: 9-, 10-, G+, 90 µs, 130 Hz, 5.0 V	Severe (25)	13	48	13	48
	21	M	27	11	Bilateral	L: 0-, 1-, G+, 120 µs, 130 Hz, 5.5 V R: 8-, 9-, G+, 120 µs, 130 Hz, 5.5 V	L: 2-, 3-, G+, 230 Hz, 125 Hz, 7.2 V R: 10-, 11-, G+, 210 µs, 125 Hz, 7.3 V	Severe (26)	16	38.5	11	57.7
	22	M	59	11	Bilateral	L: 2-, G + 60µs, 130 Hz, 1.2 V R: 10-, G+, 60µs, 130 Hz, 1.2 V	L: 2-, 3-, G+, 150 µs, 125 Hz, (4.5 – 5.5) mA R: 10-, 11-, G+, 150 µs, 150 Hz, (4.5 – 5.5) mA	Moderate (18)	11	38.9	9	50
No long-term response	23	F	35	6	Bilateral	L: 2-, 3-, G+, 90 µs, 130 Hz, 1.7V R: 10-, 11-, G+, 90 µs, 130 Hz, 1.7 V	L: 2-, 3-, G+, 90 µs, 130 Hz, 2.5V R: 10-, 11-, G+, 90 µs, 130 Hz, 2.5 V	Extreme (32)	21	34.4	26	18.8
	24	F	55	12	Bilateral	L: 2-, 3-, G + 90 µs, 130 Hz, 1.5 V. R: 10-, 11-, G + 90 µs, 130 Hz, 1.5 V	L: 2-, 3-, G+, 120 µs, 130 Hz, 5.0 mA. R: 10-, 11-, G+, 120 µs, 130 Hz, 5.0 mA	Extreme (35)	25	28.6	32	8.6
	25	F	33	10	Bilateral	L: 2-, 3-, G+, 150 µs, 130 Hz, 4.6 V R: 10-, 11-, G+, 150 µs, 130 Hz, 4.6 V	L: 2-, 3-, G+, 90 µs, 120 Hz, 5.0 V R: 10-, 11-, G+, 90 µs, 120 Hz, 5.0 V	Extreme (34)	34	0	35	-2.9 α
	26	F	32	5	Bilateral	L: 1-, 2-, G+, 60 µs, 130 Hz, 2.4V R: 9-, 10-, G+, 60 µs, 130 Hz, 2.4 mA	L: 1-, 2-, G+, 40 µs, 130 Hz, 5.0mA R: 9-, 10-, G+, 60 µs, 130 Hz, 5.0 mA	Severe (25)	10	60 β	19	24
	27	M	37	20	Unilateral (R)	R: 1-, 2-, G+, 150 µs, 140 Hz, 4.0 V	R: 1-, (4.9 mA), 2- (5.4 mA), G+, 150 µs, 140 Hz	Severe (31)	26	16.1	27	12.9
	28	F	33	5	Bilateral	L: 1-, 2-, G+, 40 µs, 149 Hz, 5.5mA R: 9-, 10-, G+, 40 µs, 149 Hz, 5.5mA	L: 1-, 2-, G+, 40 µs, 149 Hz, 5.5mA R: 9-, 10-, G+, 40 µs, 149 Hz, 5.5mA	Severe (25)	33	-32 α	21	16
	29	F	34	6	Bilateral	L: 1-, 2-, G+, 90 µs, 130 Hz, 5.5 mA R: 9-, 10-, G+, 90 µs, 130 Hz, 5.5 mA	L: 3-, 4-, G+, 90 µs, 130 Hz, 10.0 mA R: 3-, 4-, G+, 90 µs, 130 Hz, 10.0 mA	Severe (26)	29	-11.5 α	20	23.1
	30	M	34	3	Bilateral	L: 1-, 2-, G+, 60 µs, 130 Hz, 4.0 mA R: 9-, 10-, G+, 60 µs, 130 Hz, 4.0 mA	L: 1-, 2-, G+, 60 µs, 130 Hz, 4.0 mA R: 9-, 10-, G+, 60 µs, 130 Hz, 4.0 mA	Moderate (17)	12	29.4	12	29.4
Cohort summary		F 56.7% M 43.3%	40.3 ± 10.0 years	10.1 ± 5.9 years	Unilateral (R) 16.6% Bilateral 83.33%			30.2 ± 6.0 Y-BOCS Score	20.4 ± 8.2 Y-BOCS Score	32.4 ± 24.5% of reduction	14.4 ± 9.2 Y-BOCS Score	51.6 ± 29.3% of reduction

(α) Negative values shows clinical worsening of the symptoms evaluated by Y-BOCS.

(β) Patient who meet 60% of symptom reduction but not meet the 35% on the long-term period.

Patients were implanted with Medtronic model 3387 or SenSight leads, both with 1.5-mm contact spacing. Stimulation was routinely initiated within a few days after electrode implantation and progressively titrated during the first two postoperative weeks to therapeutic amplitudes, typically ranging from 1.5 to 3.0 V or mA, provided that no stimulation-related adverse effects occurred. During the first 3 months, stimulation parameters could be adjusted at intervals of approximately 4–6 weeks according to clinical response and tolerability. Thereafter, patients were generally reassessed, and stimulation parameters adjusted at approximately 3-month intervals until completion of the first postoperative year. Beyond the first year, programming and follow-up intervals were individualized according to each patient’s clinical course, therapeutic response, and tolerability.

Clinical outcomes were assessed using the Yale–Brown Obsessive Compulsive Scale (Y-BOCS), which comprises 10 items divided equally between obsession and compulsion domains. Each domain ranges from 0 to 20, yielding a total score from 0 to 40, with higher scores indicating greater symptom severity (11–13). Assessments were performed at baseline before surgery (T0), 1 year after surgery (T1), and the latest available follow-up at least 3 years after surgery (T2). T2 ranged from 3 to 21 years after baseline.

Baseline severity was categorized as extreme (Y-BOCS 32–40), severe (24–31), or moderate (16–23) (11, 12, 14). Three patients had total scores in the moderate range (one score of 17 and two scores of 18). These patients had chronic, functionally disabling predominant obsessions, documented failure of conventional treatments, and multidisciplinary approval for DBS. Their total scores reflected minimal or absent compulsions despite severe obsession-domain symptoms. The decision was consistent with a published eligibility approach that permits a lower total threshold in patients with predominant obsessions (15). Because this subgroup comprised only three patients, its outcomes were described individually and were not displayed as a group in **Figure 1**.

**Figure 1:**
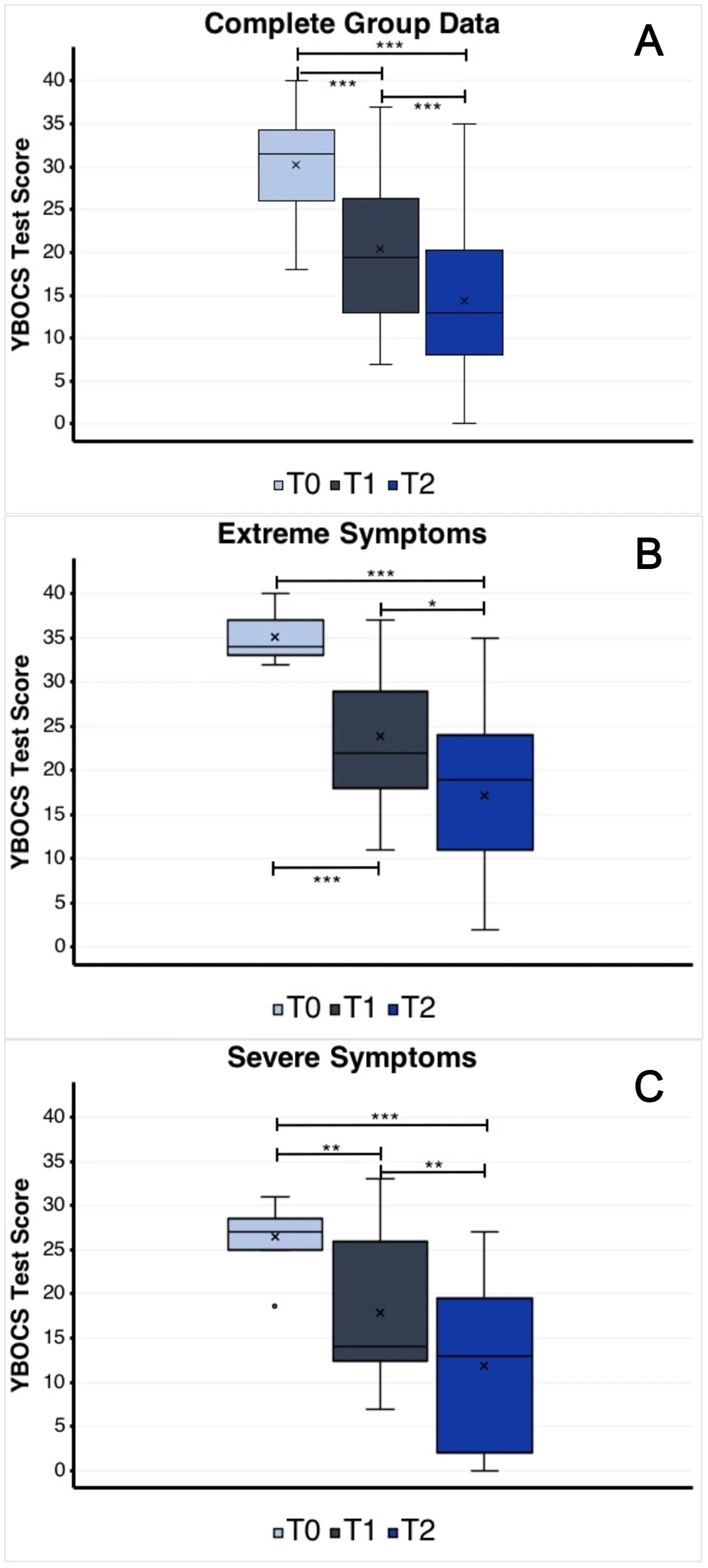
Absolute Y-BOCS scores at baseline and follow-up. **(A)** Complete cohort (n=30), **(B)** patients with extreme baseline symptoms (n=15), and **(C)** patients with severe baseline symptoms (n=12). At baseline (T0), 1 year after surgery (T1), and long-term follow-up ≥3 years (T2). The moderate subgroup (n=3) is not displayed because of its small size. Boxes represent the interquartile range, horizontal lines indicate medians, and × symbols indicate means. Whiskers show the minimum and maximum observed values. Brackets show Bonferroni-adjusted pairwise comparisons from repeated-measures ANOVA. *p<0.05; **p<0.01; ***p<0.001.

Response was defined as a ≥35% reduction in Y-BOCS score from baseline. Delayed response was defined as <35% reduction at T1 and ≥35% at T2. Early and maintained response required ≥35% reduction at both T1 and T2. No long-term response was defined as <35% reduction at T2, irrespective of the T1 result. For descriptive trajectory analyses, the delayed and early-and-maintained categories were combined as long-term responders and compared with patients without long-term response. Because these groups were defined using the T2 outcome, subgroup comparisons were considered descriptive and exploratory rather than predictive.

International consensus defines response as a ≥35% Y-BOCS reduction with a CGI-I rating of 1 or 2; partial response as a ≥25% but <35% Y-BOCS reduction with CGI-I indicating at least minimal improvement; and remission as a Y-BOCS score ≤12 with a CGI-S rating of 1 or 2, each sustained for at least 1 week (16). Because CGI-I and CGI-S were not systematically recorded in this retrospective cohort, these consensus categories could not be fully operationalized. Only the Y-BOCS component was available, and partial response and remission were not analyzed as study outcomes.

The primary analysis evaluated absolute Y-BOCS scores across T0, T1, and T2 in the complete cohort, with T0–T2 as the principal pairwise contrast. T0–T1 and T1–T2 contrasts, interval-specific percentage reductions, baseline-severity strata, and response trajectories were secondary or exploratory analyses. Percentage reduction was calculated relative to the score at the start of each interval. Repeated-measures ANOVA was performed in IBM SPSS Statistics, version 31.0 (IBM Corp., Armonk, NY, USA). Sphericity was assessed with Mauchly’s test; Greenhouse–Geisser correction was planned if the assumption was violated. For each analysis, the three pairwise comparisons were adjusted using the Bonferroni method. All statistical tests were two-sided, with the significance level set at α=0.05. 95% confidence intervals were calculated for the principal outcome estimates. Time was treated categorically because T2 occurred at heterogeneous follow-up durations, and a linear clinical trajectory was not assumed. Detailed longitudinal data on concomitant pharmacotherapy and psychotherapy were not systematically available and were not analyzed. No *a priori* sample-size calculation was performed; all eligible patients were included. Continuous variables are reported as mean ± sample standard deviation (SD). No non-parametric sensitivity analysis was performed.

## Results

The cohort comprised 30 patients, including 17 women (56.7%). Mean age at surgery was 40.3 ± 10.3 years. Mean follow-up was 10.1 ± 6.0 years (range, 3–21 years) (**Table 1**). Mauchly’s test did not indicate violation of sphericity (W = 0.885, χ²(2)=3.43, p=0.180); therefore, results under the assumption of sphericity were interpreted. Y-BOCS scores differed significantly across time points (F(2,58)=51.29, p<0.001, partial η²=0.639). Bonferroni-adjusted pairwise comparisons showed significant reductions from T0 to T1, T1 to T2, and T0 to T2 (all adjusted p<0.001; **Figure 1A**).

At baseline, 15 patients had extreme symptoms, 12 had severe symptoms, and three had moderate total Y-BOCS scores with only severe obsessions. The extreme- and severe-symptom groups showed significant improvement over long-term follow-up (**Figures 1B, C**). The moderate subgroup was not subjected to group-level inference or displayed as a boxplot because it included only three patients; their individual results are shown in **Table 1**.

At T2, 22 of 30 patients (73.3%) met the response criterion. Long-term response was observed in 12 of 15 patients with extreme baseline symptoms (80.0%), 8 of 12 with severe symptoms (66.7%), and 2 of 3 with moderate total scores (66.7%). The remaining eight patients did not meet the response criterion at T2. These response-defined subgroup analyses are descriptive because group membership was determined by the long-term outcome.

Eight patients (26.7% of the cohort) were delayed responders: five from the extreme group, two from the severe group, and one from the moderate group. A total of 14 patients showed an early and maintained response, and one patient that also met the response at T1 maintained without any change at T2.

For the complete cohort, mean Y-BOCS reduction was 32.4 ± 24.5% from T0 to T1 and 51.6 ± 29.3% from T0 to T2. Corresponding reductions were 38.5 ± 20.0% and 64.4 ± 22.5% among long-term responders and 15.6 ± 29.1% and 16.2 ± 10.2% among patients without long-term response. **Figure 2** compares the distributions of percentage reduction across the T0–T1, T1–T2, and T0–T2 intervals. In the complete cohort and among long-term responders, the magnitudes of reduction during T0–T1 and T1–T2 did not differ significantly (**Figure 2D**), whereas the cumulative T0–T2 reduction was greater than either interval-specific reduction (**Figure 2E**). No comparison was significant among patients without long-term response (**Figure 2F**).

**Figure 2:**
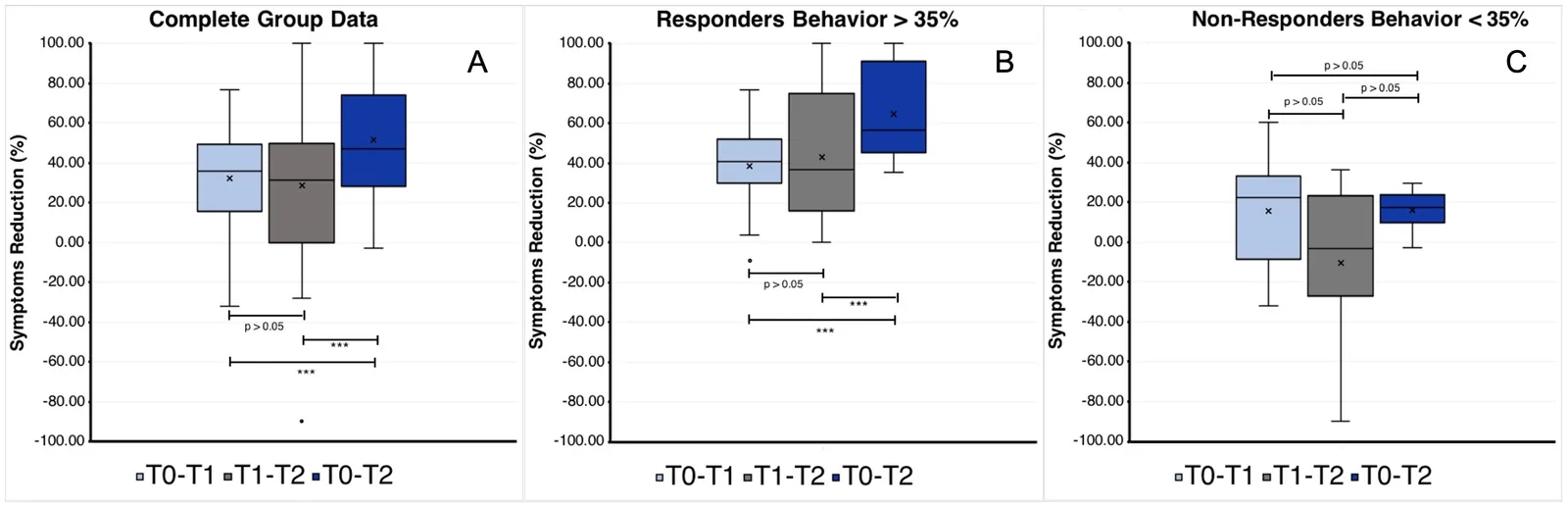
Interval-specific percentage reduction in Y-BOCS scores. Percentage reductions over T0–T1, T1–T2, and T0–T2 are shown for **(A)** the complete cohort (n=30), **(B)** long-term responders (n=22), and **(C)** patients without long-term response (n=8). Brackets compare the magnitude of percentage reduction between intervals using repeated-measures ANOVA with Bonferroni-adjusted pairwise comparisons. Boxes represent the interquartile range, horizontal lines indicate medians, and × symbols indicate means. Whiskers show the minimum and maximum observed values. p>0.05 indicates a non-significant comparison; ***p<0.001.

## Discussion

In this single-center retrospective cohort, absolute Y-BOCS scores decreased from baseline to 1 year and continued to decrease by the latest follow-up assessment. Of 30 analyzed patients, 20 met the response criterion at T2. Response was observed across baseline-severity categories, although the moderate subgroup was too small for group-level inference. These findings describe sustained improvement within the cohort but do not establish that DBS alone caused the observed long-term changes.

In comparison with the study from Graat et al. in which 50 patients were analyzed, our study followed the patient’s symptoms up to 21 years (7). Despite differences in stimulation targets, follow-up duration, and outcome definitions, our findings are consistent with previous long-term single-center and multicenter reports; however, this comparison should be interpreted cautiously because the exclusion of four operated patients without complete follow-up data may have inflated the observed response proportion. The 73.3% response rate in the analyzed cohort was higher than the pooled estimates of 66% reported by Gadot et al. and 57.9% reported by Martinho et al. (17, 18).

A clinically relevant observation was that eight patients (26.7%) who had not met the response threshold at long-term follow-up. One patient (patient 26) showed response at 1 year but did not maintain until T2. Delayed response could reflect gradual clinical adaptation, long-term optimization of stimulation settings, concomitant treatment, psychosocial changes, or natural fluctuation in OCD severity. The present retrospective data cannot distinguish among these explanations. Nevertheless, the finding supports assessment beyond 1 year before long-term outcome is judged. Among the 14 patients with an early and maintained response, 11 improved further and three remained stable. Of all 15 patients who met the response threshold at T1, and the other maintained it, indicating that subsequent loss of response was uncommon but not absent in this cohort.

The three patients with moderate total baseline scores warrant cautious interpretation. Two met the long-term response criterion, whereas one (patient 30) did not. Patient 30 underwent generator replacement one week after the T2 assessment because of reported battery-related problems and subsequently described subjective improvement. This post-T2 observation was not part of the predefined outcome analysis and requires further standardized follow-up. These three cases illustrate why total Y-BOCS scores should be interpreted together with symptom-domain severity and functional impairment, but they do not support broader conclusions about DBS in patients with moderate OCD.

Long-term trajectories also have been affected by factors not captured systematically in the dataset, including depressive symptoms, changes in medication or psychotherapy, psychosocial adversity, alcohol use, and optimization of stimulation parameters. Generator replacements were documented in patients 4, 5, 6, 17, 20, and 22; some patients reported transient symptom worsening during adaptation to new stimulation settings, including transitions between voltage- and current-controlled devices. These observations were recorded mainly in narrative clinical notes and were not accompanied by standardized Y-BOCS assessments at the time of the event. They are therefore presented as clinical context rather than quantified adverse-event outcomes. Surgical, stimulation-related, hardware, and psychiatric adverse events were not collected using a standardized framework throughout the study period, and the study cannot provide reliable long-term event rates.

The study has several limitations. Its retrospective, uncontrolled, single-center design limits causal inference and may introduce selection and information bias. Four of 34 operated patients were unavailable for long-term analysis, and their unknown outcomes may have biased the observed response proportion upward. The small sample limited precision, particularly in the exploratory severity and response subgroups. The inclusion period spanned 18 years, during which surgical techniques, hardware, and programming practices may have changed. T2 varied from 3 to 21 years and was analyzed as a categorical assessment; consequently, the analysis does not estimate a yearly rate of change or fully account for unequal observation times. Medication and psychotherapy were not systematically recorded, no continuous-time or non-parametric sensitivity analysis was performed, and adverse events were not captured comprehensively. Finally, response groups were defined using T2 and then used to describe trajectories, so these comparisons should not be interpreted as predictive analyses.

Despite these limitations, the study provides descriptive evidence that improvement after NAcc/ALIC DBS can remain clinically meaningful over very long follow-up and that some patients may meet the response threshold only after the first postoperative year. The results are most applicable to highly selected patients with treatment-resistant OCD treated in specialized multidisciplinary DBS centers. Prospective studies with standardized follow-up intervals, systematic recording of concomitant treatments and adverse events, and analyses that incorporate exact follow-up duration are needed to confirm these observations.

## Data Availability

The raw data supporting the conclusions of this article will be made available by the authors, without undue reservation.
